# A Review on *Haematococcus pluvialis* Bioprocess Optimization of Green and Red Stage Culture Conditions for the Production of Natural Astaxanthin

**DOI:** 10.3390/biom11020256

**Published:** 2021-02-10

**Authors:** Siti Nur Hazwani Oslan, Noor Fazliani Shoparwe, Abdul Hafidz Yusoff, Ainihayati Abdul Rahim, Chang Shen Chang, Joo Shun Tan, Siti Nurbaya Oslan, Kavithraashree Arumugam, Arbakariya Bin Ariff, Ahmad Ziad Sulaiman, Mohd Shamzi Mohamed

**Affiliations:** 1 Faculty of Bioengineering and Technology, University Malaysia Kelantan, Jeli Campus, Jeli 17600, Kelantan, Malaysia; fazliani.s@umk.edu.my (N.F.S.); hafidz.y@umk.edu.my (A.H.Y.); ainihayati@umk.edu.my (A.A.R.); chang.shenchang@yahoo.com (C.S.C.); ziad@umk.edu.my (A.Z.S.); 2 Bioprocessing and Biomanufacturing Research Centre, Faculty of Biotechnology and Biomolecular Sciences, Universiti Putra Malaysia, UPM Serdang 43400, Selangor, Malaysia; jooshun@usm.my (J.S.T.); arbarif@upm.edu.my (A.B.A.); 3 School of Industrial Technology, Universiti Sains Malaysia, George 11800, Penang, Malaysia; 4 Department of Biochemistry, Faculty of Biotechnology and Biomolecular Sciences, Universiti Putra Malaysia, UPM Serdang 43400, Selangor, Malaysia; snurbayaoslan@upm.edu.my; 5 Enzyme Technology Laboratory, Institute of Bioscience, Universiti Putra Malaysia, UPM Serdang 43400, Selangor, Malaysia; 6 Department of Bioprocess Technology, Faculty of Biotechnology and Biomolecular Sciences, Universiti Putra Malaysia, UPM Serdang 43400, Selangor, Malaysia; kavithraashree@gmail.com

**Keywords:** natural secondary carotenoid, *Haematococcus pluvialis*, astaxanthin, microalgae, bioprocess optimization

## Abstract

As the most recognizable natural secondary carotenoid astaxanthin producer, the green microalga *Haematococcus pluvialis* cultivation is performed via a two-stage process. The first is dedicated to biomass accumulation under growth-favoring conditions (green stage), and the second stage is for astaxanthin evolution under various stress conditions (red stage). This mini-review discusses the further improvement made on astaxanthin production by providing an overview of recent works on *H. pluvialis*, including the valuable ideas for bioprocess optimization on cell growth, and the current stress-exerting strategies for astaxanthin pigment production. The effects of nutrient constituents, especially nitrogen and carbon sources, and illumination intensity are emphasized during the green stage. On the other hand, the significance of the nitrogen depletion strategy and other exogenous factors comprising salinity, illumination, and temperature are considered for the astaxanthin inducement during the red stage. In short, any factor that interferes with the cellular processes that limit the growth or photosynthesis in the green stage could trigger the encystment process and astaxanthin formation during the red stage. This review provides an insight regarding the parameters involved in bioprocess optimization for high-value astaxanthin biosynthesis from *H. pluvialis*.

## 1. Introduction

Presently, the cellular structure of “green microalgae” bears diverse high-value metabolites that can potentially attract numerous biomanufacturing businesses [1]. *Haematococcus pluvialis* (Chlorophyceae) is a unicellular freshwater microalga with global distribution in many watery habitats and currently recognized as the richest and most promising source for the commercial production of natural astaxanthin [2,3]. Astaxanthin or (3,3′-dihydroxy-β, β-1-carotene-4,4′-dione) is a secondary carotenoid with bright blood-red color, which can be synthesized directly by exerting cellular stresses onto *H. pluvialis* [4,5]. Most astaxanthin applications are related to human nutrition and health in the form of food, pharmaceuticals, nutraceuticals, and dietary supplements [6,7].

Astaxanthin is known as the “superstar of antioxidants” due to its antioxidant capacity [8]. Additionally, it shows the importance of anti-inflammatory and antitumoral activity applications such as nutraceutical and pharmaceutical industries [9,10]. Astaxanthin is often used as an aquaculture and food colorant [2]. The supplementation of astaxanthin in aquaculture nutrition improves the growth performance, growth hormone, and the survival of Asian seabass [11]. Moreover, dietary astaxanthin provision at increasing doses markedly reduced the circulating levels of serum cholesterol and triglycerides in fish [12]. Antioxidant capacity (or the ability to terminate free radical chain reaction) of astaxanthin is 38-fold higher than that of β-carotene and 500 times stronger than vitamin E [13]. Natural astaxanthin from *H. pluvialis* as a supplement has no side effect for human consumption [4]. Thus, it is not surprising that the retail prices of nutraceutical grade astaxanthin are higher than (USD$100,000 per kg) [14], which is a value that remains valid nowadays. To date, the global market forecast up to 2025 for natural astaxanthin is estimated to hit USD 1 billion [15].

At present, the pigmentation of fish is the only practical function of synthetic astaxanthin through feed additive. Synthetic astaxanthin has not been approved as food additives and supplements for direct consumption by humans due to the difference in the molecular structure relative to the natural product [15,16]. However, synthetic astaxanthin can be synthesized through a Wittig chemical reaction under a multistep process, which includes a mixture of isomers (3S,3′S), (3S,3R), and (3R,3′R) at a ratio of 1:2:1 respectively [17,18]. During the steps before its final stage, the molecule assumes different forms when it attains the same chemical formula as natural astaxanthin. Meanwhile, the natural astaxanthin consists of (3S,3′S) [18].

Carotenoid accumulation may reach 5% of dried *H. pluvialis* biomass, of which 90% comprises of astaxanthin [19,20]. Astaxanthin is produced by *H. pluvialis* under the imposition of various environmental stresses. These include intense illumination, high/low temperature, salt stress, nutrient deprivation, and a combination of stressors to accelerate the astaxanthin production [9,21]. Pereira and Otero [14] have reported that the astaxanthin accumulation is correlated with homeostasis disruption, which induces the cells to self-protect against stresses resulted from depletion of the cellular photosynthetic capacity. The cultivation system of *H. pluvialis* mainly consists of two stages: green stage or growth phase, where they reproduce under favorable conditions to gain biomass; and the red stage resembling a non-motile red cell phase, in which the cells are placed under various stress conditions to express astaxanthin [9,22]. For that reason, simultaneously maintaining a good equilibrium between the green and red stages is vital to maximizing the astaxanthin production.

This review summarizes the most recent works on the cultivation of *H. pluvialis,* which includes strategies for the green and red stage for cultivation method. The strategies cover different strains of *H. pluvialis* with different growth conditions and process improvement subjected to stress conditions, intending to increase the astaxanthin production.

## 2. Cell Morphology of Haematococcus pluvialis

**Figure 1:**
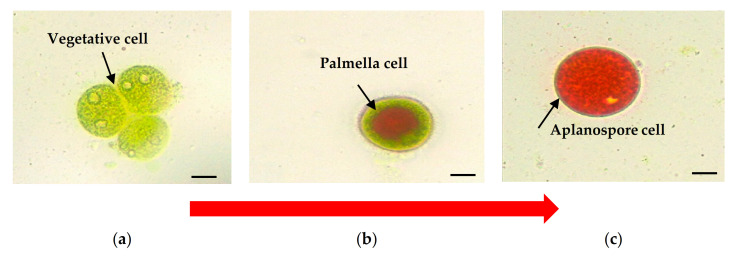
*H. pluvialis* cells photographed under the light microscope with a digital camera (Olympus B071, Olympus Optical Co., Tokyo, Japan) to observe the astaxanthin accumulation. (**a**) The vegetative stage at the beginning of the cultivation period; (**b**) the intermediate stage, wherein cells turned into greenish-orange; and (**c**) cyst stage at the end of the cultivation period; bar: 10 μm (own source).

*H. pluvialis* exposure to various stress conditions influences the cell’s ultrastructural changes throughout their life cycle [6,9]. The cell is typically spherical to an ovoid shape having a diameter of ≈30 μm. At first, *H. pluvialis* starts as a free-swimming, green biflagellate microalga with a single pyrenoid-containing chloroplast, then losing its flagella and rounding up to become a non-motile palmella, and finally transitioning to the thick-walled aplanospore [8]. The vegetative cells of *H. pluvialis* (Figure 1a), which are connected with the green stage (biomass accumulation), can asexually reproduce 2–32 daughter cells [4]. Astaxanthin starts to accumulate in the intermediate stage, beginning with the encystment process, where *Haematococcus* turns into greenish-orange cells (Figure 1b), which the transition color can be observed at 7 to 10 days. The stress conditions cause the loss of both flagella and increment of cell size. Astaxanthin is continuously being accumulated, and cells form cysts at the aplanospore stage (Figure 1c). The cells are referred to as the “red-astaxanthin formation” (red stage), where the cells are formed at 11 to 14 days. A thick algeenan-containing cell wall is formed and protects aplanospore cells from acetolysis by nutrient deprivation or high light exposure [23]. In the mature stage of aplanospores, astaxanthin accumulates densely in droplets in the perinuclear cytoplasm, resulting in the bright red color of the cells [13]. 

Most studies have shown that under favourable conditions, cells cultivation can be induced rapidly. Multiple researchers have optimized a two-stage approach to increase the mass cultivation of H. pluvialis [24,25], which can be adopted in the industry. Most of the studies have been focusing on achieving high biomass. The optimal condition for vegetative growth also needs to be optimized. It is possible to cultivate the green stage in the optimal temperature and illumination intensity to achieve maximum vegetative growth rates and biomass in a shorter period before transferring this biomass into the red stage [24,25]. Previously, strains under the vegetative stage with motile cells have had significantly higher division rates than the non-motile cells [17].

## 3. Current Production Strategy to Induce Biomass at the Green Stage

### 3.1. Effect of Nitrogen Sources

Nitrogen is one of the essential nutrients that affect cell growth and enzymatic activity of *H. pluvialis*, particularly for boosting astaxanthin production [26]. The cell growth is related to an increase in cell size, which has been observed in *H. pluvialis* [26,27]. Thus, the accumulation of biomass in the cells might also be inhibited when the cells lack nutrients during cultivation [26]. Microalgae is well known for its uptake of nitrate, ammonia, and urea as nitrogen sources [9]. The elemental composition of urea comprises approximately 46% nitrogen and complements by 20% carbon [28]. Thus, the combined availability of nitrogen and carbon favorably contributes to the increment of microalgae cells [29]. To put it into perspective, another inorganic nitrogen source frequently utilized in aquaculture media preparation, i.e., nitrate (sodium or potassium salt) only provides 16.5% of nitrogen [30]. In terms of the nitrogen uptake mechanism, the microalgae metabolism pathway could be altered due to various types of nitrogen sources. Theoretically, the mechanisms of nitrogen uptake from ammonium have the most straightforward metabolic pathway for direct assimilation by the microalgae [18]. For instance, urea, which is supplied exogenously, has a slight complex mechanism requiring an energy-driven co-transport process. Then, it needs to be reduced into ammonium ion beforehand by urease and nitrate reductase [31]. For *H. pluvialis* growth, most studies have employed the Bold Basal medium (BBM) [9]. The formulation contains inorganic nitrogen, phosphorus, and essential trace minerals, and it conforms to the need of general-purpose culture medium [20,32]. BBM ingredients initially include ammonium ion, which is favorable for the autotrophic growth of *H. pluvialis*. Nonetheless, previous work on *H. pluvialis* JNU35 cultivation found that using ammonium hydrogen carbonate (NH_4_HCO_3_) as the nitrogen source could cause medium acidification that ultimately led to cell death [9]. Yoshimura et al. [33] reported that by changing the component to urea, it is readily assimilated by urease, converted, and degraded to carbon dioxide and ammonia without producing a net acidic or basic exchange, and thus, the alkalinity of the culture remains constant. In addition, based on Wijanarko [34], the utilization of ammonia at 500 mg L^−1^ as the nitrogen source in the cultivation of *Chlorella vulgaris* could effectively increase around 55–60% of lipid formation from total cell biomass. The author proposed that it might be due to the conversion of trace ammonia into nitrate and ammonium ion, which are finally reabsorbed into the cells. Saumya et al. [29] assumed that both nitrate and ammonium ions are present in the urea-based medium under light and dark conditions, as the nitrogen source could increase the biomass accumulation even in the different trophic conditions of microalgae.

### 3.2. Effect of Carbon Sources

High *H. pluvialis* biomass can be obtained through the mixotrophic mode of cultivation utilizing acetic acid or acetate [35]. In the cultivation of *H*. *pluvialis,* the astaxanthin accumulation was induced by using an organic carbon source supplemented with 100 mM potassium acetate [13]. Sodium acetate at 30 mM was reported to improve the *H. pluvialis* cell productivity to 0.243 g L^−1^ day^−1^ [36]. Another study by Tolga et al. [37] on the cultivation of *H. pluvialis* with sodium-acetate addition (1 g L^−1^) at the beginning or the end of the log phase cultivation saw that the cell numbers in which sodium-acetate was added at the end of log-phase cultivation had increased to almost two-fold from 21.7 to 42.9 × 10^4^ cells mL^−1^. The finding showed that the biomass increased faster when compared to the addition of sodium-acetate at the beginning of the cultivation (of which the increase was only 1.2-fold). The use of sodium acetate as an organic carbon source is possible and effectual to increase the growth rate. 

The high productivity of biomass and astaxanthin could be achieved by altering the C/N balance using carbon dioxide (CO_2_), which was able to stimulate cyst formation and astaxanthin accumulation [38]. The biomass of *H. pluvialis* could be increased by carbon dioxide (CO_2_) diffusion into the media to replace the nitrogen deficiency with a specific concentration of gaseous carbon [39]. One method of achieving relative nitrogen starvation is altering the carbon/nitrogen (C/N) ratio by increasing carbon in the system as opposed to replacement with nitrogen-deficient media. Cheng et al. [40] observed the highest biomass production and astaxanthin induction with 6% CO_2_. It was hypothesized that additional carbon infusion into the culture system will shift the C/N balance and create a relative nutrient deficiency that will enhance astaxanthin accumulation. However, a further increase of CO_2_ to 20% would diminish cell growth [41]. The increase of CO_2_ level up to 20% would lead to chloroplast inhibition and decreasing cell growth, resulting in high cell mortality [39,41]. Multiple studies have recently reported on a maximum cell number of *H. pluvialis* in the region of 2.43 × 10^6^ cells/mL after providing it with sodium gluconate at 2 g L^−1^ and illumination intensity at 105 ± 3 μmol m^−2^ s^−1^ [42].

Additionally, Lu et al. [43] reported an experiment focusing on employing series gradient fed-batch strategy (SGF) whereby gradient feeding that gradually controlled the medium C/N ratio from 10 to 50 during the green stage would co-regulate the metabolism involved in cell division as well as the up-regulation of carbon assimilation for the biomass and carotenoids accumulation within H. pluvialis cells. Under this condition, the SGF strategy contributed to a hyper-density production of immotile cyst cells (final cell yield at 9.18 g L^−1^) at the end of the green stage that utilized CH_3_COONa and NaNO_3_ as the carbon and nitrogen sources, respectively. After that, subjecting the SGF grown H. pluvialis cyst cells to high light intensity treatment in the second stage eventually affected high astaxanthin productivity of 15.45 mg L^−1^ d^−1^. 

### 3.3. Effect of Illumination Intensity

Illumination is also a significant factor for improving the biomass productivity for *H. pluvialis* in mixotrophic culture [42]. Radiation energy in the life cycle of *H. pluvialis* contributes to the conversion of antenna pigments to chemical energy in the form of ATP and NADPH via a photosynthetic electron transport chain. The microalgae will store the chemical energy in starch by fixing CO_2_ through the Calvin cycle [44]. In assessing the effect of illumination and culture media toward the regulation of the *H. pluvialis* growth rate, Imamoglu et al. [45] had compared five culture media with three different light levels intensities (40, 50, 60 μmol photons m^−2^ s^−1^). Upon 12 days cultivation under low-level light intensity, *H. pluvialis* MACC-35 cells in Rudic’s Medium (RM) accumulated up to 9.50 × 10^5^ cells mL^−1^. Basal medium followed the cell growth trend to achieve 8.85 × 10^5^ cells mL^−1^, whereas the rest of the media managed to propagate the cells in the vicinity of 7.0 × 10^5^ cells mL^−1^. By merely increasing the light intensity to mid-level (50 μmol photons m^−2^ s^−1^), this then affected a 14.7% reduction in the cell concentration in RM culture to 8.10 × 10^5^ cells mL^−1^, while the observed deficit in the cell concentration was only 11% in basal medium. As expected, further reduction in the growth rate of *H. pluvialis* persisted at 60 μmol photons m^−2^ s^−1^, whereby none of the cultivation in the five media surpassed 7.0 × 10^5^ cells mL^−1^.

On the contrary, Zhang et al. [26] reported that the dry cells weight of *H. pluvialis* FACHB-712 was dramatically increased as the light intensity increased from 50 to 400 μmol m^−2^ s^−1^, which was coupled by the nitrogen depletion. The dry weight increased to 2.19 ± 0.08 g L^−1^ at the light intensity of 400 μmol photons m^−2^ s^−1^, which was 1.39 times higher than that at 50 μmol photons m^−2^ s^−1^. This event concluded that the net photosynthetic production highly depended on different light intensities. Kiperstok et al. [46] demonstrated that the production of biomass *H. pluvialis* CCAC0125 using a vertical Twin-Layer photobioreactor in cultivation at high light intensity up to 1015 μmol photons m^−2^ s^−1^ with CO_2_ supplementation in the range of 1% to 10% yielded biomass productivities of up to 19.4 g m^−2^ d ^−1^ and a final biomass of 213 g dry weight m^−2^ growth area after 16 days of cultivation.

### 3.4. Effect of Different Trophic Conditions

The cultivation of *Haematococcus* to gain biomass or astaxanthin extract is possible under photoautotrophic, heterotrophic, and mixotrophic growth mode [8,47,48]. The recipe for the photoautotrophic mode of culture typically requires light, CO_2_, water, and nutrients [49]. Light is a source of energy, whereas inorganic compound mostly serves as a carbon/nitrogen source to produce algal biomass rich in lipids, protein, and sugars [50]. Hong et al. [48] reported that a high *H. pluvialis* biomass was produced under the photoautotrophic induction process using NIES-C and NIES-N medium. In addition, the biomass concentration in cells cultured at 30 °C with ferrous sulfate was increased by 37% compared to that of cells cultured without ferrous sulfate with a value of 0.92 g L^−1^ of biomass [48].

Under the heterotrophic mode of cultivation, feeding *Haematococcus* with organic compounds in the absence of light was geared more toward increasing the cell productivity [6]. However, this technique is not really suitable for producing astaxanthin in *H. pluvialis*, since astaxanthin is a light-dependent carotenoid [47]. On the other hand, under the mixotrophic mode, the use of organic and inorganic sources for carbon and energy such as acetate-supplemented medium is successful in enhancing *H. pluvialis* growth and astaxanthin production [6,36]. However, bear in mind that the mixotrophic technique could increase cross-contamination risk by other microorganisms or grazers [8]. Wen et al. [47] reported that the mixotrophic cultivation of *H. pluvialis* in an open raceway pond is still limited due to bacterial contamination. The raceway ponds giving an effective culture area of 5 m^2^ were 2.6 m long and 2.1 m wide. However, nitrogen-depleted conditions, coupled with acetic acid addition to the cultures, would limit the reproduction of bacteria and result in more than 20% of astaxanthin productivity [47].

### 3.5. Effect of Culturing System

Recently, for the mass production of *H. pluvialis*, a few producers employed a two-step cultivation approach, either utilizing systems of bioreactors, outdoor production ponds, or the combination of both [6,15]. Firstly, small-scale green phase cells cultivated in photobioreactors are devoted to cell proliferation under normal growth conditions. Later, under stress and nutrient-deficient conditions, the cells are transferred into larger-scale raceway ponds toward astaxanthin accumulation [15]. To date, a number of works have been published pertaining to the actual and computational fluid simulation of *H. pluvialis* cultivated in different designs of photobioreactors [9,51,52]. In these reports, the outdoor cultivation of *H. pluvialis* under an enclosed photobioreactors environment was chosen as the first step. Later on, the microalgae would be exposed to the stressed condition in raceway ponds for astaxanthin production [52].

The cultivation of *H. pluvialis* by using industrial waste as a substrate can reduce industrial cultivation costs [53]. However, it is vital to control the quality of waste, since *H. pluvialis* has the ability to absorb metal ions [54]. Han et al. [55] claimed that there were some advantages over conventional cultivation methods when the green cells were inoculated and grown as biofilms. The advantages were water saving, energy saving for mixing, preventing protozoans contamination, and that they have a relatively easy harvesting technique. However, this method has only been tested indoors and might pose a significant challenge for outdoor production. Furthermore, the operational cost of biofilm photobioreactors would be severely high [46]. Thus, selecting the cultivation methodologies with current production processes and facilities are essential in reducing the operational costs and risk of contamination. In addition, it is suitable to match with the cultivation area in climatic conditions (light, temperature, rain) and an efficient system for process improvement [6].

## 4. Current Strategies Inducing Astaxanthin in the Red Stage

**Figure 2:**
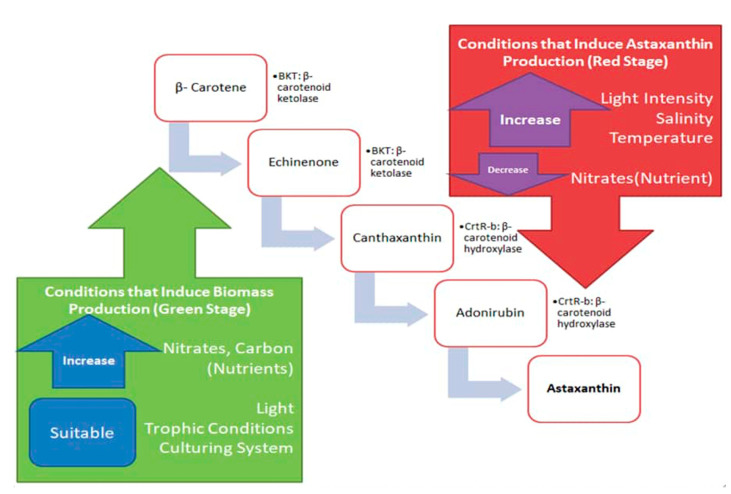
Astaxanthin formation pathway from β-carotene and conditions that induce the biosynthesis of astaxanthin in *H. pluvialis.*

Generally, astaxanthin in *H. pluvialis* starts to accumulate after introducing stress intervention in the second stage of culture in the forms of salt concentration, nitrogen depletion, varying the illumination intensity, iron concentration or increasing the temperature [22]. β-carotenoid is formed by the cyclization of red lycopene after a series of isopentenyl pyrophosphate (IPP) conversion and condensation into colorless phytoene and dehydrogenation [56]. Figure 2 summarizes the conditions responsible for inducing stress leading to the biosynthesis of astaxanthin in *H. pluvialis.* At the green stage, β-carotene, an end product for photosynthesis in plants and algae (*H. pluvialis* included) is the precursor for keto-carotenoids in the chloroplast and cytosol [57]. The oxygenation of β-carotene by β-carotene ketolase (BKT) gives rise to echinenone and canthaxanthin [58]. Under the stress condition, the multiple BKT genes are up-regulated to a certain threshold in the red stage. Then, *H. pluvialis* begin to synthesize astaxanthin [59]. Astaxanthin is synthesized from the hydroxylation of canthaxanthin catalyzed by CrtR-b in *H. pluvialis* [58].

### 4.1. Effect of Salinity

Salinity has a complex stress effect on microalgal net lipid productivity [60]. NaCl salt destroys the oxygen-evolving complex (OEC) and photosystem II (PS II) reaction centre and restrains the electron transport at its both donor and receptor sides, affecting the light energy absorption, transfer, and application, which possibly leads to algal growth inhibition and cell death [61]. Excessive extracellular inorganic ions affect the extracellular and intracellular osmotic balance, inducing exosmosis or cellular water efflux [62]. Cell death can also be related to the disruption of reactive oxygen species consumption and production equilibrium by excessive NaCl [63]. However, Gao et al. [64] concluded that distinctive salinity adaptabilities are present in different *H. pluvialis* strains, as evidently shown by the cultivation of three Australian *H. pluvialis* isolates indicating some growth even under salinity of 0.17 M (≈1%) NaCl [65]. Then, astaxanthin production cost could be reduced by applying a suitable concentration of the comparatively cheap and always in ready-stock NaCl into the microalgal variety to improve biomass, lipid, and carotenoid [61]. The formation of astaxanthin can also be induced by adding NaCl (0.25–0.5% *w*/*v*) to the media. In addition, when NaCl is added together with 2.2 mM sodium acetate, astaxanthin accumulation can be increased [32]. It has also been reported that salinity stress induces reactive oxygen species (ROS) accumulation; therefore, NaCl additions are frequently used to inhibit cell growth and stimulate the astaxanthin synthesis [66]. Tam et al. [67] tested different NaCl concentrations on both the growth and astaxanthin accumulation on *H. pluvialis*. Reduced cell growth and increased carotenoid contents per cell were observed under salinity stress.

### 4.2. Nitrogen Depletion Strategy

The hyper intracellular carotenoid or astaxanthin accumulation can be improved by subjecting culture to nutrient deficiency via nitrogen stress. There is a review stating that the effect of nitrogen deficiency in economic astaxanthin production is greater than that of light intensity [4]. However, Scibilia et al. [27] reported an experiment whereby culture grown in BG-11 medium under nitrogen starvation coupled with high light illumination at 400 μmol photons m^−2^ s^−1^ had in effect strongly induced an astaxanthin yield of 306 mg mL^−1^. The source for high light during cultivation was flourescent-based, which contributed to a 215% increment compared to a culture that was also under nitrogen starvation but with control lighting (40 μmol photons m^−2^ s^−1^). Imamoglu et al. [68] reported when nitrogen became the limiting agent, astaxanthin production was increased such as in distilled water sparged with CO_2_ and with N-free RM, which were 29.62 and 30.07 mg g^−1^, respectively. However, if too few or no nitrogen is provided, cell damage can also result from significant chlorophyll degradation. Nahidian et al. [69] in Table 1 reported that 3-fold phosphate produces the highest cell density, and the growth rate increases up to 86%. This result proves that phosphate is the most influential component for astaxanthin accumulation in *H. pluvialis*.

### 4.3. Effect of Illumination Intensity

Light stress exerted on microalgae triggers the burst of reactive oxygen species (ROS) [70]. They disrupt the cellular homeostasis and induce astaxanthin accumulation in *H. pluvialis* [14]. High light intensity is one of the most significant factors in the induction of astaxanthin accumulation, which is under moderate light conditions; for a long time, they have been associated with the induced astaxanthin production because of the depletion of nutrition in the culture medium. Lv et al. [71] had demonstrated that when light intensity increased, the Calvin cycle and TCA cycle (tricarboxylic acid cycle) provided more precursors for other pathways. The contents of various metabolites increased significantly, and astaxanthin biosynthesis also increased. According to Hu et al. [72], under light stress, the application of the nucleotides, carbohydrates, and amino acids in algal medium is associated with the astaxanthin biosynthesis. Moreover, Azizi et al. [20] reported that in a 5 L stirred-tank photobioreactor coupled with constant light intensity in the phototrophic stage, the increment of biomass and astaxanthin concentration was 50% and 60% over the BG-11 media under the constant light intensity (100 µmol m^−2^ s^−1^). In addition, Imamoglu et al. [68] also reported the influences of different light intensities on the accumulation of astaxanthin and suspension color during the induction period. In their report, the final astaxanthin concentration of 29.62 and 30.07 mg g^−1^ was obtained under high light intensity of 445 and 546 μmol photons m^−2^ s^−1^, respectively by using standard day light fluorescents lamps (18 W) positioned 2 × 2 or 3 × 3 from each side of the flasks. As the light intensity increased from 445 ^1^ to 546 μmol photons m^−2^ s^−1^, proportionately, the astaxanthin concentration in N-free medium, NP-free medium, and distilled water with sparged CO_2_ were recorded to increase by 25.5%, 15.3%, and 7.6%, respectively [68].

### 4.4. Effect of Temperature

*Haematococcus* cultures were shown to be quite sensitive to temperature changes [73]. The accumulation of astaxanthin can be generated at maximum intracellular levels of oxidative stress when optimum temperatures are exposed to *H. pluvialis*. Multiple studies reported that the maximum viable temperature for such carotenogenesis is 30 °C, and any higher temperatures than 35–40 °C will induce cell lysis in *H. pluvialis* [4,14,48]. According to Giannelli et al. [73], when *H. pluvialis* was exposed to two culture conditions at 27 and 20 °C, the high-temperature culture ultimately accumulated approximately 37% more astaxanthin than the control (156 against 115 mg L^−1^) under nitrogen starvation, with the final total cell concentration of 9.8 × 10^6^ cells mL^−1^ and 9.1 × 10^6^ cells mL^−1^ for 27 and 20 °C, respectively. The increased temperature had a positive effect when combined with the nitrogen starvation stress and allowed for increased final astaxanthin production. Furthermore, Hong et al. [48] reported that the moderate temperature (25–28 °C) improved an astaxanthin production in *H. pluvialis* compared to cells cultured in normal temperature (23 °C). The biomass concentrations in cells cultured were reduced by 20% and 48% at 30 and 36 °C under photoautotrophic induction compared to those of cells cultured at 23 °C (1.21 g L^−1^) after 18 days. The astaxanthin content in the cells cultured also decreased significantly by 31% and 62% at 30 and 36 °C, respectively compared to that of cells cultured at 23 °C (31.8 mg g^−1^) of dry weight. It was shown that heat stress from 30 to 36 °C inhibits photosynthesis in *H. pluvialis*, in which the intracellular astaxanthin is inversely related to cellular photosynthetic activity [48].

### 4.5. Effect of Metal Concentration

Recently, the potential application of metal-based nanoparticles (NPs) has been attracting applications in the cultivation of plant and algae. Multiple studies revealed the positive effects of metal-based NPs on certain algae when appropriate concentrations were applied to the cultures [74,75]. He et al. [76] showed that the cell density and chlorophyll content were enhanced at 20 mg L^−1^ supplementation of Fe_2_O_3_ NPs in the growth medium of *Scenedesmus obliquus*. Kadar et al. [77] revealed that by adding Zn NPs at 1.17 × 10^−5^ M concentration in the growth medium of microalgae, the growth rate was stimulated. Rastar et al. [75] indicated that a positive influence of biomass, astaxanthin, and chlorophyll contents of *H. pluvialis* was obtained in the presence of 2.49 and 4.41 mg L^−1^ Zn NPs as per their standard concentration in BBM medium. However, higher concentrations of the metals NPs may lead to toxic effects, resulting in the cell density of *H. pluvialis* significantly decreasing, due to its restriction on the microalgae for the light accessibility and finally disturbing the photosynthesis process. Similarly, Djaeramane et al. [74] reported that the treatment of ZnO NPs on *H. pluvialis* at the concentration of 10–200 μg mL^−1^ resulted in the reduction on the cell viability, biomass, and photosynthetic pigments together with surface and intracellular damages. In other microalga systems, Sibi et al. [78] showed that by increasing the CuNPs, Pb-NPs, Zn NPs, and Mg-NPs concentrations in the growth media, the specific growth rate and biomass density of *Chlorella vulgaris* were significantly decreased. Hence, some metal-based NPs, such as Fe NPs, have the potent toxicity by generating a reactive form of oxygen species (ROS), which induces the oxidative stress. Excess ROS production is believed to induce oxidative damage to the microorganism cell walls and DNA [75]. Table 1 tabulates some of the current strategies in inducing astaxanthin production during the red stage, encompassing nitrogen depletion, salinity effect, illumination intensity, temperature effect, and application of metal concentrations.

## 5. Conclusions

In conclusion, astaxanthin produced by *H. pluvialis* has created a high value-added metabolite with some unique benefits in various industrial sectors. In this review, the laboratory-scale microalgae cultivation elaborated the processes under environmental stresses to induce astaxanthin in the red stage and favorable conditions for biomass production in the green stage. *H. pluvialis* cultivation could be sustained in biomass and astaxanthin production with current cultivation strategies. Most studies have shown that BBM medium is suitable for cultivating green microalgae started with an initial biomass of 0.4 to 0.5 g L^−1^. The optimum ranges of temperature and pH for *H. pluvialis* at the green stage are 20 to 25 °C and pH 6 to 8, respectively. In addition, cultivation conditions under low light intensity, with white plasma light source under photoperiod 12:12 h light/dark cycle coupled with urea as the nitrogen source are the best combination for the growth of the cells. In comparison to the red stage, the optimal condition to induce astaxanthin production is by applying stresses such as nitrogen depletion, salinity effect, illumination intensity, temperature effect, and application of metal ion concentration. However, this summary leads to a great deal of confusion when attempting to compare results for different strains or isolate between laboratories where different species may have different optimal green and red stage parameters. Perhaps, selective combinations of techniques for two-stage microalgae cultivation will affect a much higher yield of astaxanthin to be used in various industrial applications.

## Figures and Tables

**Table 1:** Current strategies in inducing astaxanthin production in different strain of *Haematococcus pluvialis.*

	Optimal *Haematococcus pluvialis* Growth Conditions(Green Stage)					Optimal Stress Condition for Inducing Astaxanthin Production by *Haematococcus pluvialis* (Red Stage)		References
Basal Medium	Inoculum (Days)	Temp (°C)	pH	Light Intensity	Time Course (Days)	Stress Condition	Effect	
Strain JNU35 Modified BBM (mBBM) Modified BG-11 (mBG-11)	7 days Initial biomass 0.4–0.5 g L^−1^	25 ± 1	6–7	150 μmol photons m^−2^s^−1^	15	**Different Initial Nitrogen:** Sodium nitrate (NaNO_3_) Ammonium bicarbonate (NH_4_HCO_3_) Or urea ((NH_2_)_2_CO)	Best N source: Urea Max biomass (10.2 g L^−1^) Max Astaxanthin (5.4 mg L^−1^)	[9]
Strain ZY-18 NIES-C	Initial biomass 0.4 g L^−1^	25	7.5–8	250 μmol photons m^−2^s^−1^ 12 h:12 h Light: Dark cycle	until achieved 10 g L^−1^ of biomass	**Temperature:** Daytime temperature range (8 °C to 33 °C) Night temperature (maintained at 28 °C) Night temperature range(8 °C to 33 °C) Daytime temperature(maintained at 28 °C)	Daytime temperature (23 to 28 °C) is best for photoinduction, and the night temperature should be kept below 28 °C. The net biomass and astaxanthin productivities under the controlled temperature (2.34 g m^−2^ d^−1^) (60 mg m^−2^ d^−1^) were 5-fold and 2.9-fold, while of those under the natural temperature (biomass: 0.47 g m^−2^ d^−1^; astaxanthin: 21 mg m^−2^ d^−1^), respectively.	[79]
Strain Flotow RM	Initial cell 6 × 10^4^cell mL^−1^	25	NR	1.5 klux density 12 h:12 h Light: Dark cycle	15	**Different salinity** 0.8%, 1.5% and 2.5% NaCl	Astaxanthin increased 4.8 folds from 10 pg⋅cell^−1^ to 48 pg⋅cell^−1^ at 2.5% NaCl under high temperature.	[67]
Strain K-0084 BG-11	5 × 10^5^ cell mL^−1^	22 ± 1	NR	16 h:8 h Light: Dark cycle	10	**Nitrogen starvation and high light intensity**	High light (400 μmol photons m^−2^s^−1^) combined with nitrogen starvation is the most effective condition to induce astaxanthin production	[27]
Strain com-mercial MLA medium	5% (*v*/*v*) inoculum with 4.07 × 10^4^ cell mL^−1^	20 ± 1.5	NR	Photon flux density 65–75 μE m^−2^ s ^−1^ 14 h:10 h Light: Dark cycle	17	**Nitrogen depletion** Culture were grown autotrophically and undergo natural exhaustion of nitrate.	The cell size increased within the cell population, which the cell diameter average ≈30% and the cell density decreased during senescence.	[80]
Strain NIES-144; UTEX-2505 BG-11	Initial density1 × 10^4^ cell mL^−1^	NR	7–7.5	50 μmol photons m^−2^s^−1^(White LED) 12 h:12 h Light: Dark cycle	9	**Different nutrient and light-feeding strategy** Nutrients: MgSO_4_·7H_2_O, H_3_BO_3_ Na_2_CO_3_	Biomass 0.15 g L^−1^d^−1^ Astaxanthin 13.33 mg L^−1^d^−1^ Utilizing RSM technique of under constant light intensity.	[20]
Strain NIES-144 Kobayashi basal medium	The initial cell biomass ~1.0 g L^−1^	25 ± 1	7.5	4 ± 1 μmol photons m^−2^s^−1^, provided by cool white fluorescent tubes	12	**Different ratio of carbon to nitrogen (C/N)**	Biomass 9.18 g L^−1^ (100% immotile cyst cells) Astaxanthin productivity 15.45 mg L^−1^d^−1^	[43]
Strain (Isolate, Iran) BBM	Initial cell number 2 × 10^5^ cell mL^−1^	25 ± 1	NR	20 μmol photons m^−2^s^−1^ 16 h:8 h Light: Dark cycle	15	**Different macro/micronutrients** Nitrate and phosphate (macronutrients) Iron and boron(trace elements)	The modified BBM with 3-fold higher phosphate led to the highest cell density and up to 86% increase in the growth rate.	[69]
Strain Flotow EGE MACC-35 BG-11	Seven-day-old culture of green cells about 0.26 mg mL^−1^	25 ± 1	<8.0	100 µmol photons m^−2^s^−1^	14	**Different stress media with different light intensity** Rudic’s medium (RM) Nitrogen-free RM medium (N-free) Phosphate-free RM medium (P-free) Nitrogen and phosphate-free RM medium (NP- free) and Distilled water with the sparging of CO_2_ **Light Intensity** 445 and 546 μmol photons m^−2^s^−1^	Astaxanthin concentrations: Distilled water with CO_2_ (29.62 mg g^−^^1^) N-free RM medium (30.07 mg g^−^^1^) at 546 μmol photons m^−2^s^−1^	[68]
Strain SAG 19-a BBM	Initial cell 4 × 10^5^cell mL^−1^	25 ± 1	NR	Under fluorescent light	15	**Effect of the four variables** Carbon dioxide 1.54% Sodium nitrate 1.06 g L^−1^ Inoculum volume 24.97% Light intensity 2.42 klux	Positive effect on cell growth leading to maximum yield of dried biomass at 0.51 g L^−1^	[81]
Strain SAG 19-a Basal medium	4-day old culture Inoculum 4.95 × 10^5^ cell mL^−1^	25 ± 1	7	Under a continuous light intensity of 1.5 klux	12–16	**Effect of salinity with added sodium acetate (2.2 mM)** Range 0.25, 0.5, 1.0, and 2.0% *w*/*v* **Effect of nitrogen source with 0.25%NaCl and sodium acetate (4.4 mM)** Calcium nitrate; potassium nitrate; ammonium nitrate; sodium nitrate **Effect of pH with added sodium acetate (4.4 mM)** pH 5–9	Astaxanthin content was higher in acetate supplemented medium, in which an increment was obtained at 0.25 and 0.5% salinity. The maximum cell concentration was obtained in potassium nitrate (6.2 × 10^5^ cell mL^−1^) and the lowest was obtained in ammonium nitrate (1.65 × 10^5^ cell mL^−1^) grown cultures. There was a significant increase in astaxanthin productivity in media at pH 6–8.Older cells accumulated 8.3–10.69 mg L^−1^ astaxanthin compared to 0.95–8.1 mg L^−1^ in 4–8-day-old cultures, respectively.	[32]

NR = Not reported; LED = Light-emitting diode; RSM = Response Surface Methodology.
